# A bibliometric analysis of global research status and trends in irritable bowel syndrome and gut microbiota metabolites

**DOI:** 10.3389/fmicb.2025.1559926

**Published:** 2025-08-04

**Authors:** Shi-Ran Wang, Jie Zhou, Jia-Yi Zhang, Shi-Fang Li, Guo-Jie Hu

**Affiliations:** ^1^Department of Traditional Chinese Medicine, The Affiliated Hospital of Qingdao University, Qingdao, Shandong, China; ^2^Qingdao Medical College, Qingdao University, Qingdao, Shandong, China; ^3^Department of Neurosurgery, Affiliated Hospital of Qingdao University, Qingdao, China

**Keywords:** gut microbiota, gut microbiota metabolites, IBS, bibliometric analysis, research progress, research trend

## Abstract

**Background:**

Metabolites derived from the gut microbiota are substantial contributors to the pathophysiology of Irritable Bowel Syndrome (IBS). As our understanding of the mechanisms underlying gut microbiota metabolites advances, significant progress has been made in research exploring the correlation between gut microbiota metabolites and IBS. Nevertheless, a comprehensive synthesis of research foci and trends in this domain is still lacking. Consequently, integrating scientometric methods with a range of analytical tools can facilitate the identification of key research areas and potential future study directions.

**Methods:**

The present study employed scientometric tools, including VOSviewer, Bibliometrix software, CiteSpace, Tableau, and R software, to collect and analyze research literature on IBS and gut microbiota metabolites. This comprised an analysis of journal publications and their impact, the identification of prolific authors, the establishment of national research collaboration networks, and the co-occurrence analysis of keywords.

**Results:**

The analysis revealed that, following screening, a total of 1,489 documents were obtained, with a gradual increase in the number of publications starting from 2006. The United States, China, and the United Kingdom have been identified as the primary hubs of this research field. The leading research institutions were identified as University College Cork, the Mayo Clinic, and the University of California. In the domain of research under discussion, John F. Cryan, Timothy G. Dinan, and Gerard Clarke are the most prominent authors. Multiple analyses of the keywords revealed that research on gut microbiota metabolites in irritable bowel syndrome primarily focuses on the functions and mechanisms of action of specific metabolites (1). Emerging research hotspots on gut microbial metabolites influencing IBS are centered on bile acids. In contrast, chain fatty acids have been the most studied metabolites in past research. (2) Gut microbiota metabolites primarily affect IBS through the critical role of the gut-brain axis and are closely associated with anxiety-like behaviors. (3) Experimental types related to gut microbiota metabolites and IBS research.

**Conclusion:**

This study employed bibliometric analysis to map the knowledge structure and identify research hotspots in gut microbiota metabolites and IBS, providing insights for future studies.

## 1 Introduction

IBS is the most prevalent form of functional gastrointestinal disorder, characterized by a constellation of gastrointestinal symptoms that encompass abdominal pain, bloating, distension, and irregular bowel movements (Sebastián Domingo, 2022). Empirical studies have indicated that 11% of the global population is afflicted by IBS (Canavan et al., 2014). IBS has been demonstrated to exhibit a strong positive correlation with the levels of anxiety and depression among patients. Conversely, elevated anxiety and adverse emotional states have been shown to intensify the symptoms experienced by patients, thus creating a vicious cycle (Aljahdli et al., 2024). The condition imposes a considerable financial burden on individuals and exacerbates the economic strain on society and the healthcare system (Bazarganipour et al., 2020; Snijkers et al., 2024). However, the etiological mechanisms remain to be fully elucidated. Consequently, this has resulted in a limitation of the range of available therapeutic interventions for IBS. In recent years, a growing body of research has demonstrated that gut microbiota metabolites play a crucial role in the pathogenesis of IBS (Shrestha et al., 2022).

The gut microbiota, a complex ecosystem comprising bacteria, fungi, and viruses, is located within the intestinal tract. A healthy gut microbiota is characterized by diversity and equilibrium; however, in individuals with IBS, this equilibrium is often perturbed (Menees and Chey, 2018). It has been demonstrated that specific bacterial groups are strongly linked to the development of IBS (Chong et al., 2019). Perturbations within the intestinal microbiota, characterized by a reduction in beneficial bacterial populations and a concurrent increase in harmful bacteria, may precipitate the symptoms of IBS (Wang et al., 2023). Gut microbiota metabolites are associated with diarrhea-predominant symptoms in IBS patients. Dysregulation of bile acid metabolism is closely related to the pathogenesis of IBS (Chong et al., 2019). Moreover, abnormalities in tryptophan metabolism are intimately linked to the underlying pathophysiology of IBS (Clarke et al., 2009; Chojnacki et al., 2023). Butyric acid, a key short-chain fatty acid, alleviates IBS symptoms through enhancing intestinal barrier function, exerting anti-inflammatory effects, and modulating gut microbiota (Shaidullov et al., 2021; Gargari et al., 2023; Jiang et al., 2022). This underscores the pivotal role of gut microbiota metabolites in the pathogenesis of IBS. Although studies have been conducted on gut microbiota metabolites and the mechanism of IBS, the variety of gut microbiota metabolites and the complexity of their mechanism of action have not yet been fully elucidated, and this knowledge does not meet the therapeutic needs (Cheng et al., 2024).

Bibliometric analysis, employing mathematical and statistical methodologies, provides a quantitative analysis of literature within a research domain. This approach delineates the interrelations among scholarly publications, the influence of researchers and institutions within a specific field, and highlights the focal points, priorities, and cutting-edge advancements of the research area (Ahmad and Slots, 2000; Garg et al., 2024). In recent years, as research into IBS has intensified, studies have increasingly focused on the role of gut microbial metabolites in the pathogenesis of the disease. However, systematic synthesis of research hotspots and trends within this domain is scarce. To deepen our understanding of this research field, our study employs bibliometric analysis to examine three decades of literature, revealing trends and directions in clinical and scientific research related to gut microbial metabolites in IBS, and providing theoretical and data support for subsequent studies.

## 2 Methods

### 2.1 Literature resource search strategy and selection criteria

This search was conducted to retrieve literature data related to the research field under investigation within the Web of Science Core Collection (WoSCC), a multidisciplinary, comprehensive database recognized for its comprehensive citation indexing. During this period, the following term combinations were searched: terms related to Irritable Bowel Syndrome: TS = (“irritable bowel syndrome*” OR “IBS”); and terms related to the microbiome, namely: TS = (“microbiome” OR “microflora” OR “microbiota” OR “flora” OR “probiotic” OR “Saccharomyces” OR “Lactobacillus” OR “Bifidobacterium” OR “*Escherichia coli*”); as well as terms related to gut microbiota metabolites, TS = (“metabolite*” OR “metabolism” OR “metabolome” OR “metabolite products” OR “metabolites” OR “intestinal metabolites” OR “bile acids” OR “primary bile acids” OR “secondary bile acids” OR “bile salts” OR “conjugated bile acids” OR “SCFAs” OR “short-chain fatty acids” OR “acetate” OR “propionate” OR “butyrate” OR “tryptophan” OR “neurotransmitters” OR “GABA” OR “dopamine” OR “5-HT” OR “serotonin” OR “vitamins” OR “vitamin D” OR “vitamin B6” OR “hypoxanthine” OR “hypoxanthines” OR “fatty acids” OR “amino acids” OR “ammonia” OR “amines” OR “phenols” OR “indoles”). Specific genera and specific gut flora metabolite terms were added to provide a more comprehensive and less likely to miss relevant articles. These microbial terms are derived from relevant reviews of the intestinal flora (Tian et al., 2017; Zyoud et al., 2019; John et al., 2018; Memba et al., 2017; Linares et al., 2016), and the search strategy for this section on the intestinal flora is outlined in the published articles. These gut flora metabolites all play a role in IBS (Xiao et al., 2021). The literature search spanned from January 1993 to November 2024 to ensure a comprehensive review and capture emerging trends. This search produced a total of 1,559 documents.

**Figure 1:**
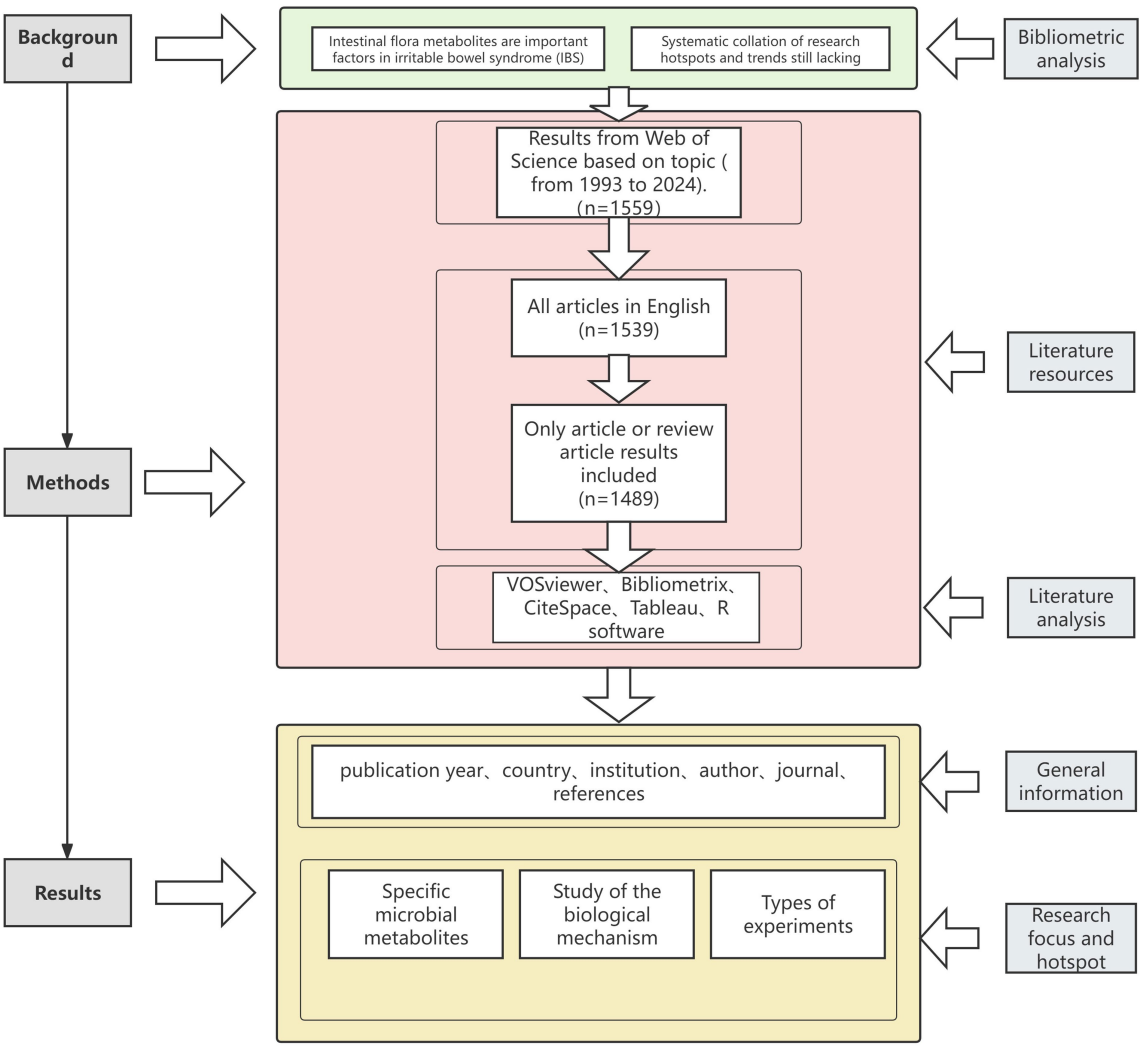
Research process.

Inclusion criteria included the following: (1) studies published between 1993 and 2024 (as of November 10); (2) studies belonging to the category of “articles” or “review articles”; and (Aljahdli et al., 2024) studies published in English only. After excluding 20 non-English articles and 50 articles that did not meet the inclusion criteria, a total of 1,489 papers related to metabolites of intestinal flora were obtained (Figure 1). The raw data were exported to “txt” format and then imported into the bibliometric analysis software for analysis. Through these procedures, we obtained a total of 1,489 journal articles for the analysis of research hotspots. We systematically collected and analyzed the core bibliographic information of the documents, including titles, authors, affiliations, countries, abstracts, keywords, publication journals, publication years, references, and citation counts. The purpose of organizing and studying these elements is to provide a comprehensive academic overview and to understand the influence of the literature within the field. This will facilitate a deeper understanding of the hotspots in the field of gut microbial metabolites and IBS, as well as the relevance of academic attention and current research trends.

### 2.2 Bibliometric analysis tools and methods

This study employs bibliometric methods to analyze research frontiers and hotspots in the field of gut microbial metabolites and IBS. Data analysis and management were performed using VOSviewer (v1.6.20), CiteSpace (v6.4. R1), Tableau (2024.3.0), R software (v4.4.1), and Microsoft Office Excel 2021. Tableau was used for mapping international collaboration networks, while VOSviewer visualized highly co-cited literature and patterns of keyword co-occurrence. An online analytical platform^1^ and R software were used to generate national collaborative networks, journal publication trends, and multidimensional linkage networks connecting institutions, authors, and countries. Microsoft Excel facilitated data management, annual publication tallies, and the generation of tables. CiteSpace enabled detailed visualizations for institutional, authorial, and keyword analysis. This integrated approach significantly enhanced analytical precision and visual interpretability of findings. The research workflow is illustrated in Figure 1.

## 3 Results

### 3.1 Analysis of annual publications and trends in publications

**Figure 2:**
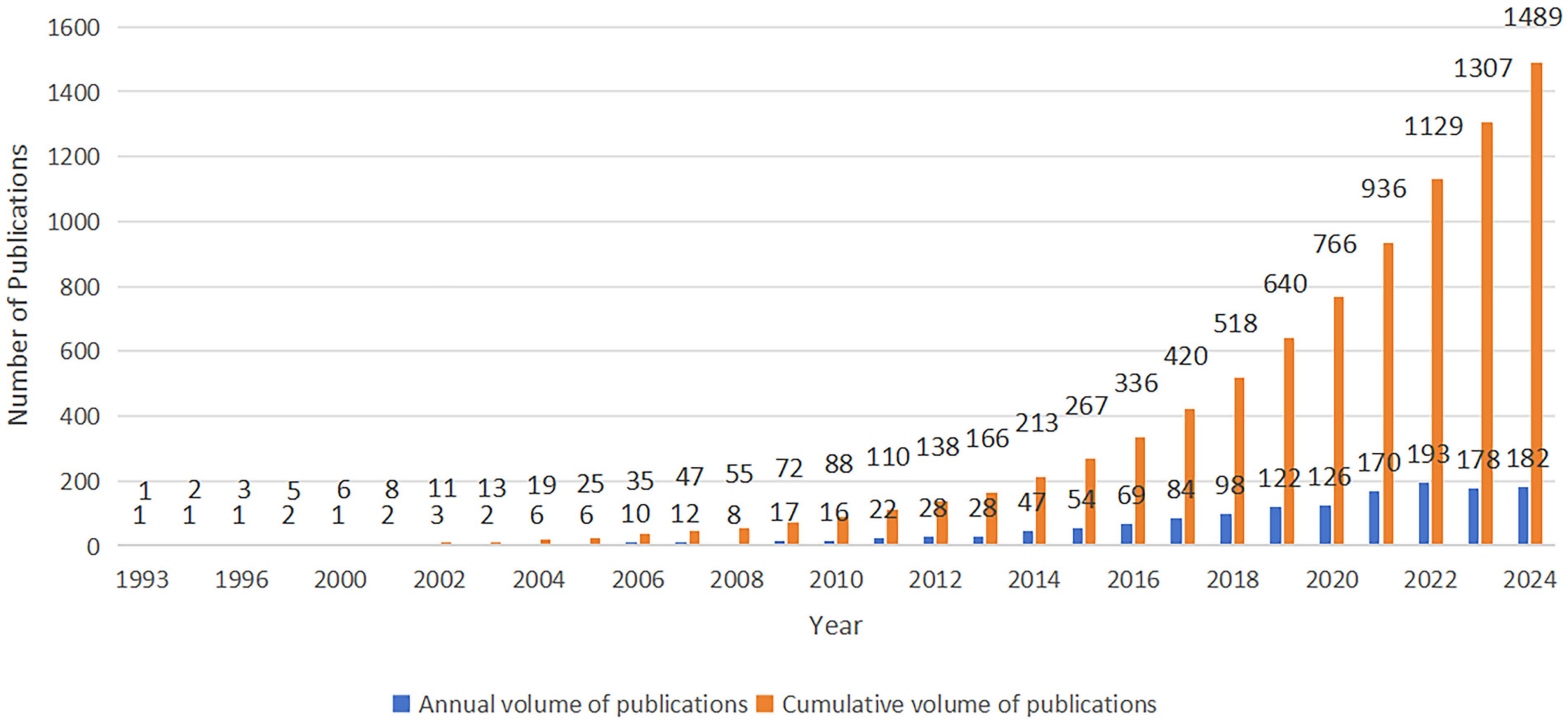
The number of annually published articles and the cumulative number of published articles.

To provide a comprehensive review of the development trends in research on gut microbiota metabolites and IBS, an analysis was conducted of the annual publication output in this field from 1993 to 2024 (Figure 2). The consistent upward trend in the number of publications indicates significant attention from academic researchers and substantial progress in this area. Between 1993 and 2005, the field was in its early stages, characterized by a relatively low number of publications. However, from 2006 to 2020, a marked growth phase in research was observed, with a particularly notable increase after 2010. The increase in publications, from fewer than 6 (1993–2005) to 16 (2010), reflects the growing interest after 2010. This surge is concomitant with the emergence of microbiome research (Sakowski et al., 2019) and advancements in research technologies (Cullen et al., 2020). Significant improvements have been made in techniques and methodologies for studying the gut microbiota, and the collective progress in societal and scientific technologies has fostered the development of medical research. The number of new publications reached a peak in 2021, with 170, and in 2022, with 193, demonstrating the research fervor in this field. Despite a modest decline in the number of new publications in 2023 and 2024, the figures remained substantial. This analysis suggests that as research becomes more intricate, its complexity may increase substantially, potentially leading to a relative deceleration in the pace of progress.

### 3.2 Analysis of the trend and collaborations of countries, institutions, and authors

**Figure 3:**
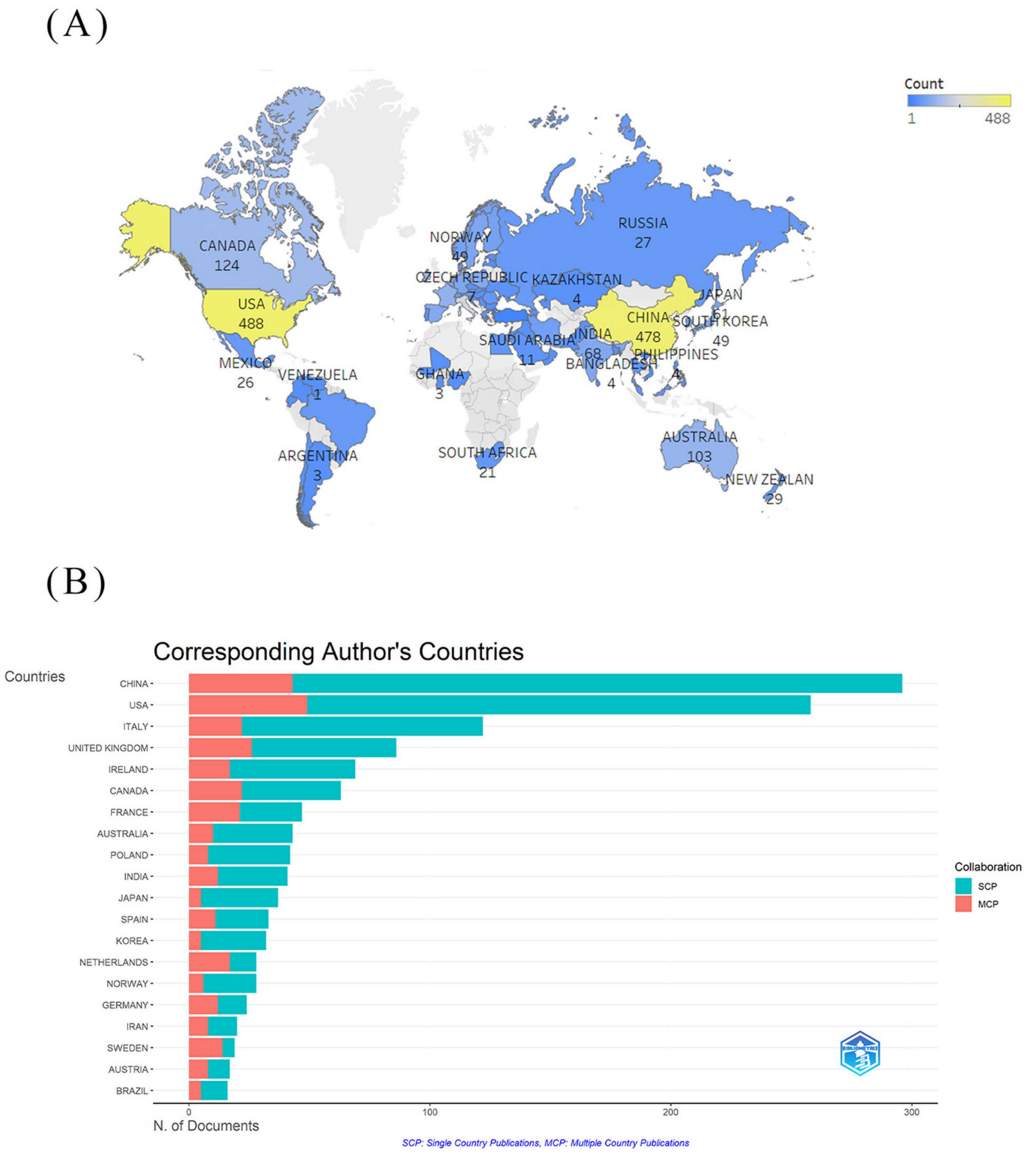
National cooperation network diagram. **(A)** National visualization network map. **(B)** Single country publications (SCP measures the domestic collaboration intensity or academic self-sufficiency of a country) and multiple country publications (MCP measures the intemnationdlcolgnoration intensity of a country) of the 20 countries with the highest production.

Within the domain of gut microbiota metabolites and IBS research (Table 1), the United States has published the most articles, with a total of 488 publications. This demonstrates the country’s dominant position in this specific research area. China is a close second with 478 publications, indicating a rising presence and significance in this research area. The United States has the highest centrality of 0.36, signifying its influential role and citation rate within the field. The United Kingdom, with a centrality of 0.33, reflects its high-impact research, slightly below that of the United States. Figure 3A illustrates the global distribution of publications on gut microbiota metabolites and IBS research. This visualization reveals that North America, Europe, and Asia are the primary regions for research in this field. Figure 3B differentiates between Single Country Publications (SCP) and Multiple Country Publications (MCP). MCP quantifies a country’s publications involving at least one foreign co-author, reflecting its capacity for international collaboration. SCP measures publications with exclusively domestic co-authors, indicating a country’s domestic collaboration strength or academic self-reliance. China is significantly ahead in SCP, indicating its active engagement and leadership in IBS and microbial metabolite research. Conversely, the United States is distinguished by its notable involvement in MCP, signifying its significant contributions to international collaborative efforts.

**Table 1:** Top 10 countries with the highest productivity on gut microbiota metabolites and IBS.

Rank	Countries	Publications	Centrality	Area
1	United States	488	0.36	North America
2	China	478	0.09	Asia
3	Italy	225	0.13	Europe
4	England	174	0.33	Europe
5	Canada	124	0.08	North America
6	Ireland	118	0.03	Europe
7	Australia	103	0.07	Oceania
8	France	100	0.08	Europe
9	Netherlands	82	0.13	Europe
10	Spain	74	0.06	Europe

**Figure 4:**
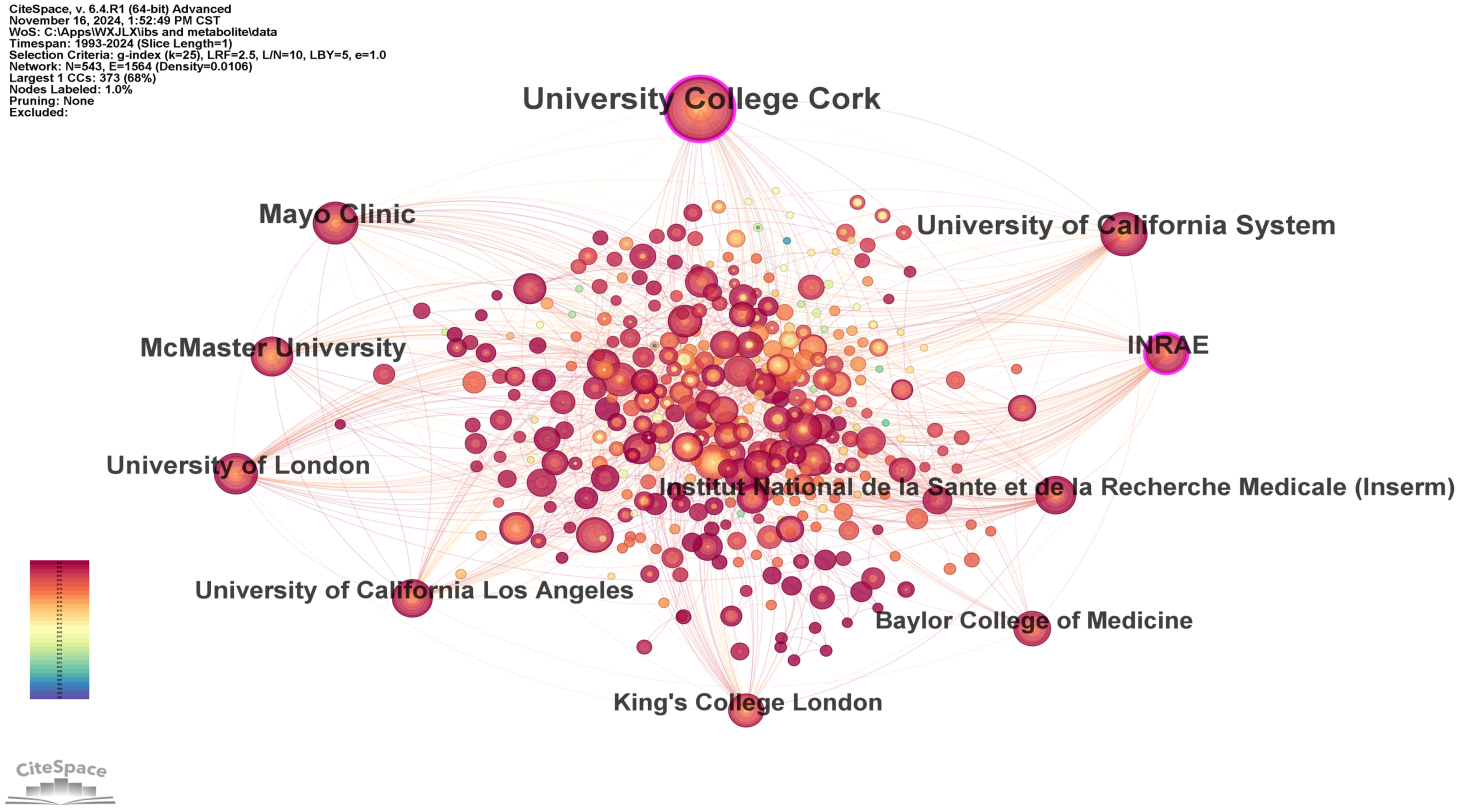
The main collaboration network of institutions.

In the domain of gut microbiota metabolites and IBS research, our analysis of the top 10 research institutions by publication output (Table 2), along with their centrality and countries of origin, reveals significant contributions from various institutions worldwide. University College Cork leads with 115 publications, underscoring its prominent role in the field. Mayo Clinic follows with 58 publications, securing the second position. The centrality of University College Cork, at 0.18, is the highest among the institutions, suggesting its research has a substantial impact and citation rate within the domain. INRAE, with a centrality of 0.11, ranks second, indicating a notable influence in the research area. The scientific collaboration network visualized through CiteSpace software provides an intuitive representation of the cooperation patterns among research institutions. In Figure 4, University College Cork, Mayo Clinic, and the University of California System have larger nodes, demonstrating their research output and influence in the field. A direct collaboration line between University College Cork and the Mayo Clinic indicates a strong research partnership. Furthermore, a majority of the research institutions are indicated by red nodes, suggesting that the research was conducted recently.

**Table 2:** The top 10 institutions with high publications on gut microbiota metabolites and IBS.

Rank	Institutions	Publications	Centrality	Country
1	University College Cork	115	0.18	Ireland
2	Mayo Clinic	58	0.09	United States
3	University of California System	57	0.09	United States
4	McMaster University	52	0.09	Canada
5	INRAE	48	0.11	France
6	University of London	43	0.1	United Kingdom
7	Institut National de la Sante et de la Recherche Medicale (Inserm)	40	0.07	France
8	University of California Los Angeles	39	0.04	United States
9	Baylor College of Medicine	35	0.01	United States
10	King’s College London	34	0.06	United Kingdom

**Figure 5:**
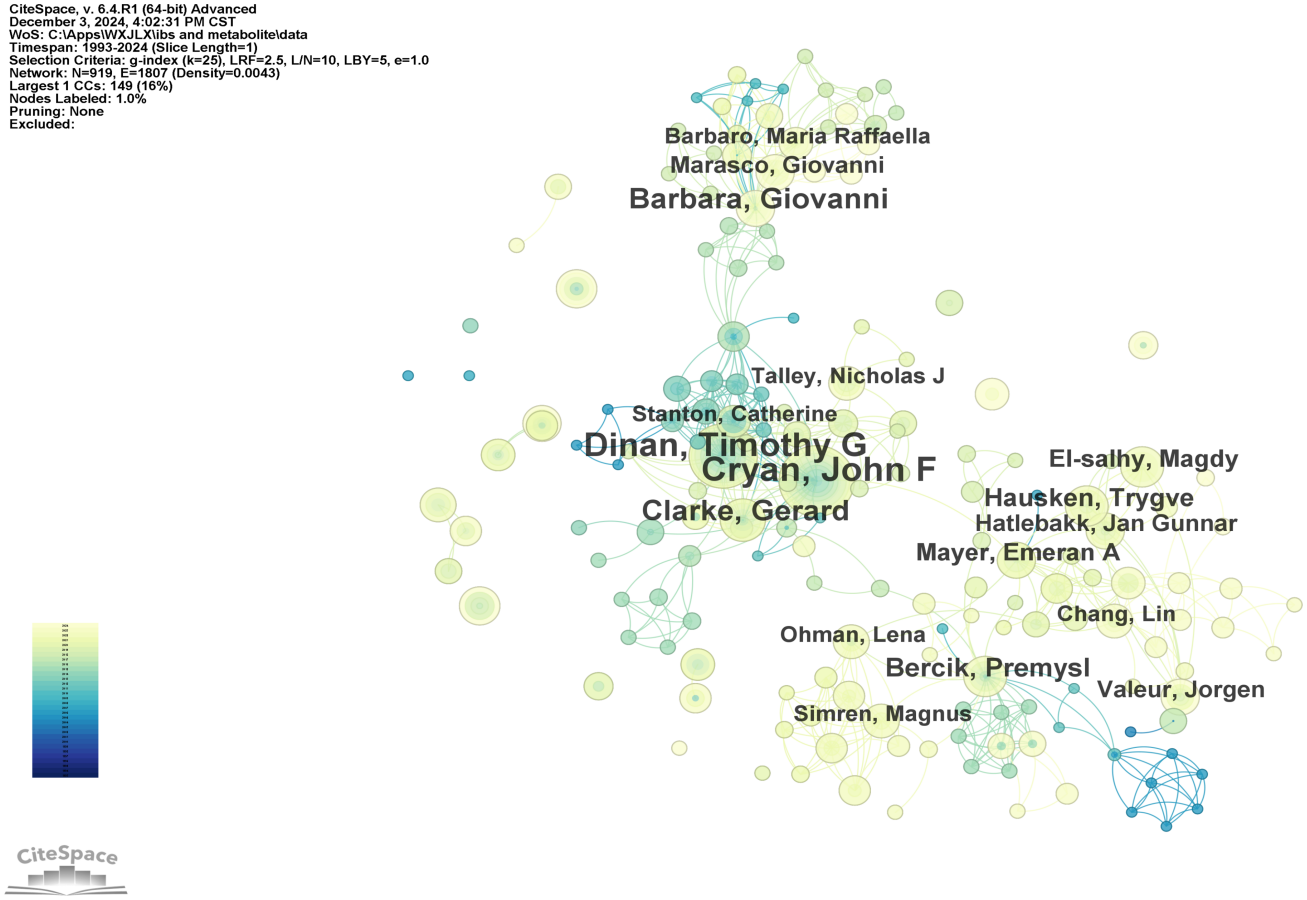
The collaboration network of core authors.

In the domain of gut microbiota metabolites and IBS research, the top 10 authors have been identified based on publication output (Table 3), along with the years of their publications and the number of papers they have authored. It is evident that Cryan, John F., has made a substantial contribution to the field, with 55 papers published, thus establishing his leadership role in the area. Dinan, Timothy G., and Clarke, Gerard follow with 47 and 28 papers, respectively, highlighting their importance in IBS and microbial metabolite research. Hausken, Trygve, and El-Salhy, Magdy, despite having fewer papers, have more recent publication years (2019 and 2020), indicating their emergence as leaders in the field. Camilleri, Michael, with 18 papers in 2008, demonstrates sustained research activity in the area. The author collaboration network generated by CiteSpace software offers a visual representation of the collaborative relationships among the main researchers in this field. As illustrated in Figure 5, prominent researchers, represented by larger nodes, such as Cryan, John F., Dinan, Timothy G., and Clarke, Gerard, are indicative of their substantial research output and influence within the field.

**Table 3:** The top 10 most productive authors on gut microbiota metabolites and IBS.

Rank	Authors	Year	Papers
1	Cryan, John F	2009	55
2	Dinan, Timothy G	2009	47
3	Clarke, Gerard	2009	28
4	Barbara, Giovanni	2009	26
5	Bercik, Premysl	2010	19
6	Hausken, Trygve	2019	18
7	Camilleri, Michael	2008	18
8	El-salhy, Magdy	2020	16
9	Mayer, Emeran A	2007	15
10	de vos, Willem M	2017	15

**Figure 6:**
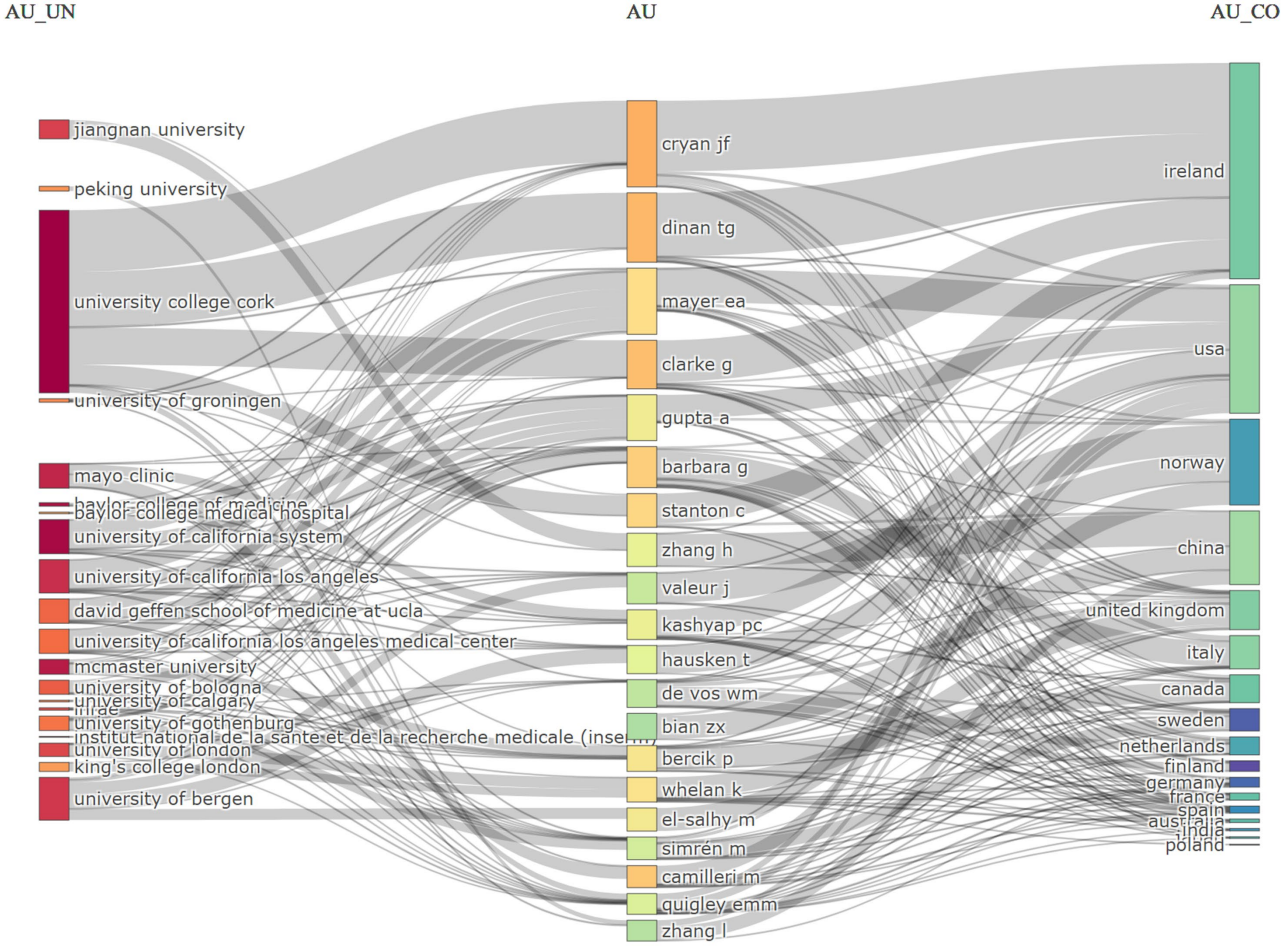
A study of the interconnections between institutions, authors, and nations.

The network of collaborations among institutions, authors, and countries in the field of IBS and gut microbiota metabolites research is illustrated in Figure 6. Prominent institutions such as University College Cork, Baylor College of Medicine, the University of California, Los Angeles, and Jiangnan University are highlighted for their significant research output and influence in collaborations. Ireland, the United States, Norway, and China stand out for their contributions to IBS and gut microbiota metabolites research, with notable collaborations, particularly between Ireland and University College Cork, as well as notable authors such as Cryan, John F., and Dinan, Timothy G.

### 3.3 Most influential journals in the field of gut microbiota metabolites and IBS research

**Figure 7:**
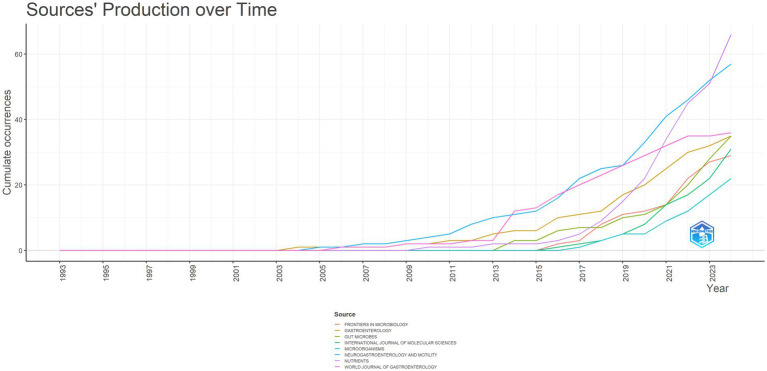
Trends in journal publication in the field of irritable bowel syndrome and gut microbiota metabolites.

In the realm of IBS and gut microbiota metabolites research, the most prestigious academic journals have been identified based on their 2023 Impact Factor (IF2023) and citation counts (Table 4). The following journals have been identified as the leading publications in this field: GASTROENTEROLOGY, with 1817 citations and an impact factor of 26.3, tops the list, indicating its significant influence in the field. GUT follows with 1711 citations, reflecting its focus on gastroenterology and its relevance to IBS and gut microbiota metabolites research. PLOS ONE, recognized for its multidisciplinary science approach and its inclusion of studies related to IBS and gut microbiota metabolites, ranks third with 1,441 citations. Figure 7 illustrates the cumulative publications in different academic journals from 1993 to 2024 in the IBS and gut microbiota metabolites research field. A notable increase has been observed in Neuroendocrinology and Metabolism since 2011, suggesting a potential correlation between gut microbiota metabolites, IBS, and the emerging focus on research in the fields of neuroendocrinology and metabolism.

**Table 4:** The top 10 most productive journals on gut microbiota metabolites and IBS.

Rank	Citations	Full journal title	IF2023	WOS Categories
1	1817	Gastroenterology	26.3	Gastroenterology and Hepatology
2	1711	Gut	23.1	Gastroenterology and Hepatology
3	1,441	PLoS One	2.9	Multidisciplinary Sciences
4	1,364	Neurogastroenterology and Motility	3.5	Clinical Neurology; Gastroenterology and Hepatology; Neurosciences
5	1,323	World Journal of Gastroenterology	4.3	Gastroenterology and Hepatology
6	1,320	Alimentary Pharmacology and Therapeutics	6.6	Gastroenterology and Hepatology; Pharmacology and Pharmacy
7	1,315	American Journal of Gastroenterology	8.5	Gastroenterology and Hepatology
8	1,252	Nature	50.5	Multidisciplinary Sciences
9	1,140	Proceedings of The National Academy of Sciences of The United States of America	9.4	Multidisciplinary Sciences
10	1,081	Scientific Reports	3.8	Multidisciplinary Sciences

### 3.4 Analysis of co-cited references

**Figure 8:**
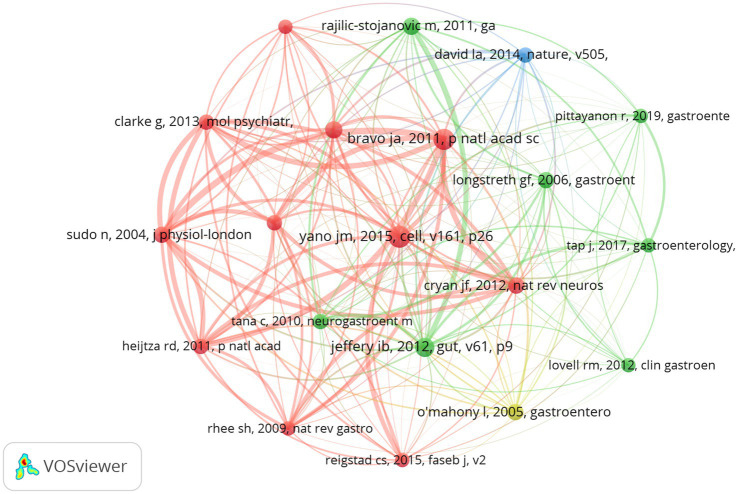
Visualization of a clustering map of co-cited references. Created with VOSviewer.

Co-cited references are those cited collectively by researchers. Through the analysis of co-cited references, VOSviewer visualizes the co-cited references, highlighting common research areas between Gut Microbiota Metabolites and IBS (Figure 8). According to VOSviewer’s results, a total of 79,800 references were cited in this research area. When the minimum number of citations in the cited literature is set to 101, 20 meet the criteria. The co-cited literature on IBS reveals distinct research clusters, with the red cluster emerging as the most prominent. This cluster encompasses core themes in gut-brain axis research (Clarke et al., 2013), including neurobiological mechanisms (Bravo et al., 2011), the bidirectional relationship between gut microbiota and stress responses (O’Mahony et al., 2009; Sudo et al., 2004), and the regulation of serotonin synthesis by gut microbiota metabolites (Reigstad et al., 2015; Yano et al., 2015). A seminal study within this cluster, “Ingestion of Lactobacillus strains modulates emotional behavior and central GABA receptor expression via the vagus nerve in mice” has garnered significant attention for its pivotal contributions to understanding gut microbiota metabolites in IBS pathophysiology (Bravo et al., 2011). Its frequent citation underscores the critical role of gut bacteria in mediating gut-brain communication, particularly through vagus nerve signaling and modulation of neurotransmitters. In contrast, the green cluster focuses on characterizing gut microbiota composition (Rajilić-Stojanović et al., 2011) and its implications for IBS subtyping (Jeffery et al., 2012). This cluster prioritizes investigations into gut microbiota metabolites as etiological factors, emphasizing their potential to inform diagnostic classifications and mechanistic models of IBS (Tana et al., 2010). The yellow cluster is associated with clinical research on IBS, where some papers explore diagnostics, symptoms, and therapeutic approaches (O’Mahony et al., 2005). The blue cluster represents research discussing molecular mechanisms or specific treatment modalities for IBS. Lawrence A. David et al.’s research suggests that the increased abundance and activity of *Bilophila wadsworthia* on animal-based diets support a link between dietary fat, bile acids, and the outgrowth of microorganisms capable of triggering inflammatory bowel disease (David et al., 2014).

### 3.5 Analysis of keywords co-occurrence, clustering, and burst

**Figure 9:**
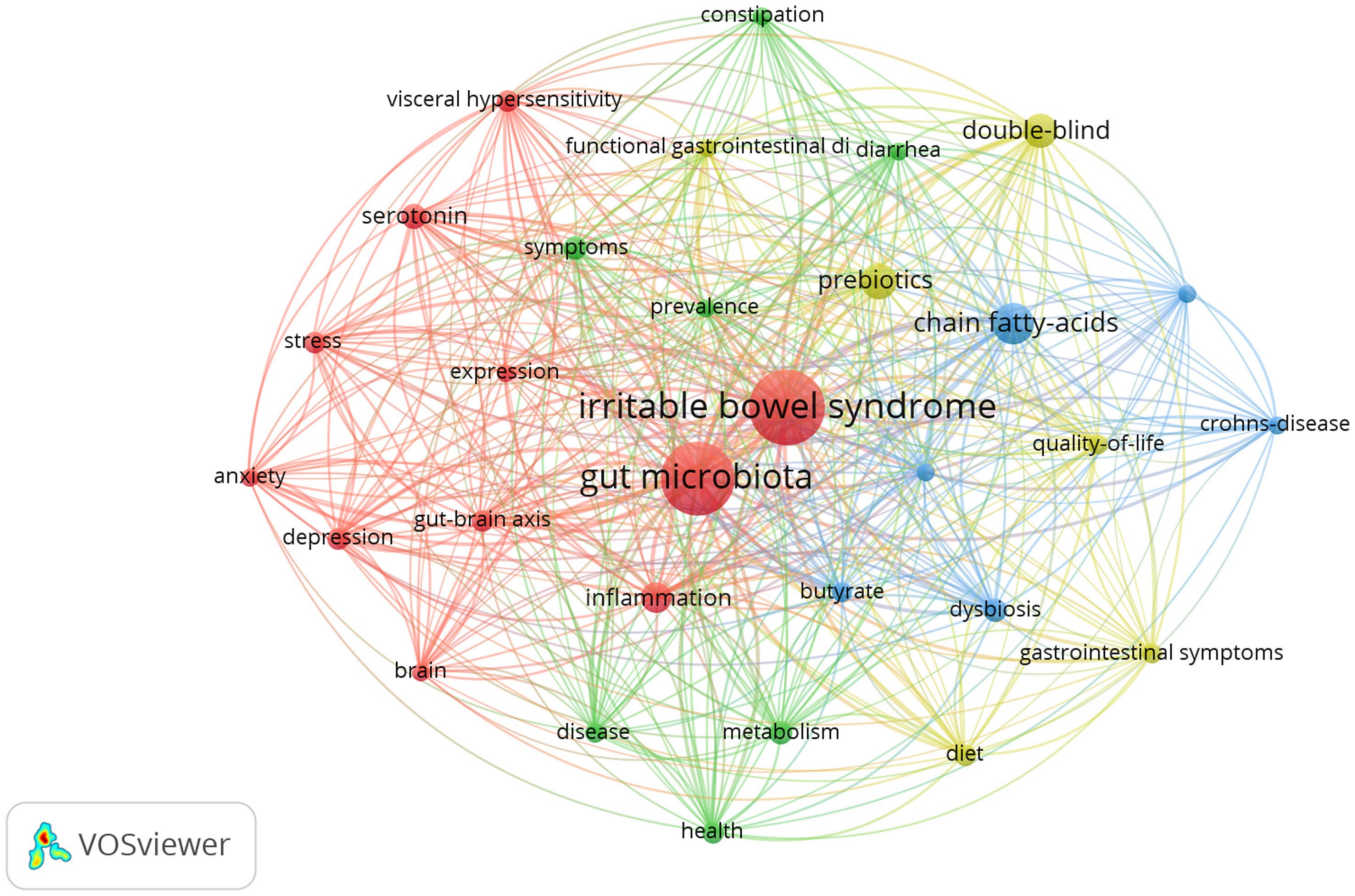
Keyword co-occurrence map of gut microbiota metabolites and IBS. Created with VOSviewer.

In our analysis of scholarly works on gut microbiota metabolites and IBS, we identified and performed a frequency analysis of keywords, resulting in the top 10 most recurrent terms, as depicted in Table 5. The table indicates that “irritable bowel syndrome,” “gut microbiota,” and “chain fatty acids” are frequently highlighted, suggesting the significance of gut microbiota metabolites such as chain fatty acids. The aspects related to the pathophysiology of IBS, including “inflammation,” “gut-brain axis,” “metabolism,” and “visceral hypersensitivity,” are frequently mentioned. Furthermore, the frequent mention of “symptoms” and “health” underscores the research community’s focus on the clinical manifestations of IBS and its impact on patient well-being. “double-blind” is frequently mentioned, indicating that this is the predominant experimental paradigm in studies examining the effects of gut microbiota metabolites on IBS. Furthermore, “double-blind” ranked first in terms of centrality, followed by “chain fatty acids,” “bacteria,” “gastrointestinal symptoms,” “ulcerative colitis,” “irritable bowel syndrome,” and “gut-brain axis.” As shown in Figure 9, these keywords combine irritable bowel syndrome and gut microbiota metabolites with other studies.

**Table 5:** Top 10 keywords in terms of frequency and centrality on gut microbiota metabolites and IBS.

Rank	Keywords	Frequency	Keywords	Centrality
1	Irritable bowel syndrome	1,601	Double blind	0.09
2	Gut microbiota	1,361	Chain fatty acids	0.07
3	Chain fatty acids	441	Bacteria	0.07
4	Double blind	314	Gastrointestinal symptoms	0.07
5	Inflammation	159	Ulcerative colitis	0.06
6	Symptoms	155	Irritable bowel syndrome	0.05
7	Metabolism	144	Gut-brain axis	0.05
8	Health	137	Functional gastrointestinal disorders	0.05
9	Gut-brain axis	131	Metabolism	0.04
10	Visceral hypersensitivity	122	Inflammatory bowel disease	0.04

**Figure 10:**
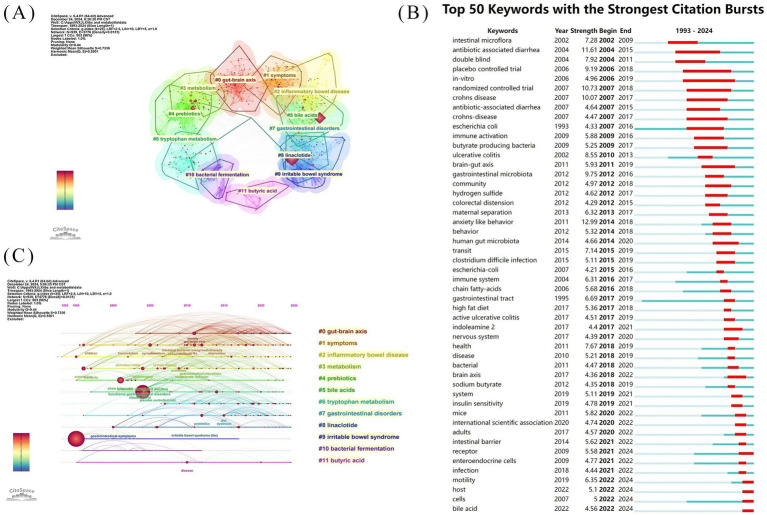
Keyword analysis diagram of IBS and gut microbiota metabolites research **(A)** Cluster analysis of co-cited keywords of IBS and gut microbiota metabolites research **(B)** Keywords with the strongest citation bursts. **(C)** Time-zone evolution diagram of IBS and gut microbiota metabolites research topics.

To delineate the research frontiers in the field of gut microbiota metabolites and IBS since 1993, we utilized CiteSpace to cluster keywords associated with gut microbiota metabolites and IBS (Figures 10A,C). Cluster #0 is labeled “gut-brain axis,” followed by Cluster #1, labeled “symptoms,” Cluster #2 “inflammatory bowel disease,” Cluster #3 “metabolism,” Cluster #4 “prebiotics,” Cluster #5 “bile acids,” Cluster #6 “tryptophan metabolism,” Cluster #7 “gastrointestinal disorders,” Cluster #8 “linaclotide,” Cluster #9 “irritable bowel syndrome,” Cluster #10 “bacterial fermentation,” and Cluster #11 “butyric acid.” These clusters indicate the predominant themes of research conducted since 1993.

Keyword bursts sum up the sudden growth of research content over time which may indicate future trends in research. Figure 10B Shows the 50 keywords with the strongest outbreaks in the domain. The blue lines represent the time intervals, and the red lines represent the duration of the keyword bursts. The thematic evolution of the keywords has evolved from “intestinal microflora” and “antibiotic-associated diarrhea” to “brain-gut axis”, “immune system”, and “chain fatty-acids”, culminating in the current focus on “intestinal barrier”, “motility”, and “bile acids.” the highest burst keywords include “anxiety-like behavior”, “antibiotic-associated diarrhea”, with the burst strength of 12.99 and 11.61. The term “double-blind” not only has a high burst value of 7.92 in keyword highlighting but also has the highest centrality (Table 5). In addition, the keyword bursts for “randomized controlled trials” and “placebo-controlled trials” were also high at 10.73 and 9.19, respectively. “*In vitro*” demonstrates the longest sustained keyword prominence at 13 years, exceeding all other terms in burst duration

## 4 Discussion

### 4.1 General information discussion

Employing bibliometric methodologies, this study provided a comprehensive analysis of the research trends concerning IBS and its association with gut microbiota metabolites. Our analysis, covering the period from 1993 to 2024, reveals a notable surge in research interest, which correlates with the burgeoning field of microbiome research and an enhanced understanding of the pathophysiology of IBS. Key contributors in this field are John F. Cryan, Timothy G. Dinan, and Gerard Clarke. The United States, China, and the United Kingdom are recognized as leading research hubs. The United States and China are demonstrating a significant lead in publication output compared to other nations. The number of papers published in the United States was 488, and the number of publications in China was 478. Additionally, University College Cork in Ireland, the Mayo Clinic, and the University of California are recognized as principal research institutions. GASTROENTEROLOGY, with 1817 citations and an impact factor of 26.3, tops the list, indicating its significant influence in the field. High-citation clusters of papers are predominantly concentrated in four areas: the gut-brain axis and neurobiological mechanisms, intestinal microbiota and gut microbiota metabolites, diagnostics, symptoms, and therapeutic approaches for IBS, and molecular mechanisms or specific treatment strategies for IBS (Figure 8).

### 4.2 Research focus and hotspot

Bibliometric analyses can reflect the hotspots and frontiers in this research field. Through multiple analyses of co-citations and keywords in the literature, we found that the hotspots and trends in gut microbiota metabolites and IBS were primarily related to the types of metabolites, their functions, and the mechanisms of action. The hotspot gut microbiota metabolites include “chain fatty acids,” “bile acids,” “butyric acid,” and “tryptophan metabolism.” The centrality of short-chain fatty acids (SCFAs) as metabolites with the highest keyword frequency (441 times) and centrality (0.07) (Table 5) stems from their widely demonstrated multiple physiological functions, including maintenance of the intestinal barrier and anti-inflammatory effects (Shaidullov et al., 2021; Gargari et al., 2023; Jiang et al., 2022). According to the keyword burst analysis, bile acids are an emerging research hotspot in gut microbiota metabolites. Its keyword explosion is from 2022 to 2024. The association between intestinal flora metabolites and irritable bowel syndrome involves multiple mechanisms, including “gut-brain axis,” “metabolism,” “inflammation,” and “visceral hypersensitivity.” Keyword co-occurrence, clustering, and co-citation mapping together strongly corroborate the “gut-brain axis” as a central hub connecting flora metabolites to IBS pathophysiology.

### 4.3 Emerging hotspots and mechanism exploration in the study of metabolites of intestinal flora

Accumulating evidence suggests that gut microbiota metabolites have a significant impact on IBS. These metabolites modulate host neurological functions through the gut-brain axis, consequently influencing both host health and IBS progression (Barandouzi et al., 2022; García Mansilla et al., 2024).

#### 4.3.1 Bile acids and irritable bowel syndrome are closely related

Bibliometric analyses enabled us to understand that the emerging mechanism linking gut microbiota metabolites to IBS is related to bile acids, which we will discuss in detail. Bile acids are amphipathic molecules synthesized by the liver that emulsify dietary lipids within the intestinal lumen to facilitate digestion and absorption. As prosecretory agents, bile acids stimulate intestinal fluid secretion while modulating gut motility through vagal nerve stimulation. Additionally, bile acids modulate visceral sensitivity by lowering visceral sensory thresholds, potentiating abdominal pain and discomfort in IBS patients (Min et al., 2022). Bile acids regulate intestinal and hepatic metabolic functions by activating signaling pathways mediated through the Farnesoid X receptor (FXR) and G protein-coupled receptors (GPCRs) (Ramírez-Pérez et al., 2017). Gut microbiota play a crucial role in bile acid metabolism through the bioconversion of primary to secondary bile acids, a process essential for maintaining intestinal environmental stability (Cheng et al., 2024; Min et al., 2022). IBS-D patients exhibit elevated Clostridia levels in their gut microbiota, potentially accelerating bile acid biosynthesis and enterohepatic cycling, which can reduce gastrointestinal transit time and exacerbate diarrheal manifestations (Xiao et al., 2021; Zhan et al., 2020). FXR agonists not only improve bile acid metabolism but also have anti-inflammatory and intestinal flora-regulating effects, providing a new direction in the treatment of IBS (Yang et al., 2024). Although bile acids have been identified as a key emerging research hotspot, a notable gap in current research is that, although studies have shown abnormal bile acid metabolism to be strongly associated with IBS-D, research on other subtypes remains limited. Abnormal bile acid metabolism is present in approximately 30% of patients with IBS-D, whereas bile acid metabolism in patients with IBS-C has not been characterized (Min et al., 2022; Camilleri et al., 2023).

#### 4.3.2 Short-chain fatty acids are the most studied metabolites of gut flora in irritable bowel syndrome

Short-chain fatty acids (SCFAs) play a significant role in IBS. These compounds protect the intestinal mucosa from inflammatory processes characteristic of inflammatory bowel disease via multiple pathways. Specifically, butyric acid suppresses the release of pro-inflammatory cytokines and regulates immune responses by activating GPCRs (Zhao et al., 2024). Another experimental study showed that SCFAs regulate the synthesis and release of 5-hydroxytryptamine (5-HT), which plays an important role in the pathophysiological process of IBS, by acting on enterochromaffin cells, and that a decrease in SCFAs leads to abnormal levels of 5-HT, which in turn triggers the symptoms of IBS (Mars et al., 2020). However, we believe that we should not only study a single metabolite, but should link them together. The current research deficiency lies in the fact that the complex synergistic and antagonistic relationships between gut flora metabolites have not been fully explored (Min et al., 2022; Chen et al., 2024; Wilson et al., 2023). The next research direction may be to study the joint mechanism of action of the gut flora metabolite network in IBS.

### 4.4 Bidirectional microbial metabolite-brain-gut axis regulatory mechanisms and psychobehavioural associations

It was found that the effects of gut microbiota metabolites on IBS are not limited to the local gut environment, but also mediate central nervous system function through the gut-brain axis. Bibliometric analyses revealed that the brain-gut axis was a significant component in both keyword clustering and co-citation analyses. In addition, ‘anxiety-like behavior’ was the most intense keyword in the keyword highlighting, with an intensity of 12.99 (Figure 10B). Consistent with previous studies, Epigenetic findings also suggest that environmental factors, such as physical and psychological stress, as well as changes in gut flora, play a crucial role in the clinical presentation of IBS (Gazouli et al., 2016). Gut microbiota metabolites can modulate mood and cognitive functions with direct signaling to the brain via the vagus nerve (Jiang et al., 2024; Kyei-Baffour et al., 2025). Animal experiments demonstrate that secondary bile acids inhibit the NLRP3-mediated inflammatory vesicle in macrophages through the activation of TGR5 receptors and thereby ameliorate metabolic syndrome (Di Giorgio et al., 2025). Vagotomy blocks its anti-inflammatory and metabolic regulatory effects, confirming a key role in nerve signaling (Liu et al., 2016). Gut microbiota metabolites can also modulate stress responses and emotional states by influencing the hypothalamic–pituitary–adrenal axis (Lu et al., 2024; Ortega et al., 2023). Preclinical studies demonstrate that kynurenine metabolites modulate prefrontal serotonin (5-HT) release through NMDA receptor-dependent mechanisms, thereby inducing cognitive impairment (Lim et al., 2017). In clinical applications, brain-gut behavioral therapies encompass cognitive behavioral therapy, relaxation training, hypnotherapy, and other interventions. These methods can reduce the symptoms of IBS patients by regulating the activity of the central nervous system (Goodoory et al., 2024). However, gut microbiota metabolites exert complex modulatory effects on mental behavior via the gut-brain axis. Specifically, short-chain fatty acids exhibit neuroprotective properties, whereas lipopolysaccharide-induced inflammatory responses contribute to hippocampal neuron dysfunction and neuronal death (Batista et al., 2019). The effects of such conflicting roles have yet to be studied.

### 4.5 Types of experiments related to gut microbiota metabolites and IBS research

Based on multiple analyses of keyword burst intensity and keyword frequency, and centrality, it was found that “double-blind,” “randomized controlled trials,” “placebo-controlled trials,” and “*in vitro*” are keywords with high scores. This highlights the importance of evidence-based medicine. A double-blind, randomized, crossover-controlled trial investigated the effects of FODMAPs in patients with IBS (Nordin et al., 2024). Paineau et al. implemented a double-blind, placebo-controlled trial enrolling 105 IBS patients who were administered 5 g/day of short-chain inulin-type fructans. The intervention demonstrated statistically significant improvement in IBS Symptom Severity Score compared to placebo controls (Menees and Chey, 2018). However, key limitations have also been exposed: existing randomized controlled trials generally suffer from small sample sizes and short follow-up periods, making it difficult to assess the long-term effects of interventions and hindering clinical translation (Mars et al., 2020). *In vitro*,” the word with the longest duration of emergence, is indispensable in mechanistic exploration. Still, there are limitations to its ability to predict the effects of complex environments in the human body.

### 4.6 Limitations of the study

Our findings, while comprehensive, are still limited by methodological and contextual factors. Relying primarily on the Web of Science Core Collection as a data source may omit regionally indexed literature, potentially biasing the analysis of the global research landscape. Additionally, the language restriction limited to the inclusion of English language literature may marginalize non-English language studies that contribute to the critical importance of gut flora metabolites in irritable bowel syndrome. Future studies should employ multi-platform searches with expanded languages to compensate for these shortcomings.

## 5 Conclusion

Employing bibliometric analysis, this study systematically examines the intellectual framework and research focal points within the domain of IBS related to gut microbial metabolites. Current evidence suggests, the functions and mechanisms of gut microbial metabolites in the etiology of irritable bowel syndrome and the development of new therapeutic strategies targeting the gut microbiota constitute key areas of research. The findings of this research provide a data-driven foundation and theoretical benchmark for future research initiatives in this field.

## Data Availability

The raw data supporting the conclusions of this article will be made available by the authors, without undue reservation.

## Footnotes

^1^Bibliometrix; https://www.bibliometrix.org
